# Peripheral Primitive Neuroectodermal Tumor: A Rare Case in Pediatrics

**DOI:** 10.7759/cureus.39005

**Published:** 2023-05-14

**Authors:** Atef A Rashed, Reem Alharthi, Shuaa Aljabri, Raghad Alsubhi, Deemah H Bukhari

**Affiliations:** 1 Pediatrics, Maternity and Children’s Hospital, Makkah, SAU; 2 Medicine and Surgery, Umm Al-Qura University, Makkah, SAU; 3 Otolaryngology - Head and Neck Surgery, Maternity and Children’s Hospital, Makkah, SAU

**Keywords:** liver tumor, primitive neuroectodermal tumor, peripheral primtive neuroectodermal tumor, pnet, ppnet

## Abstract

Primitive neuroectodermal tumors (PNETs) are a type of malignant tumors made up of small neuroectodermal-derived round cells that affect soft tissue and bone, with a wide range of clinical symptoms and histological commonalities depending on the site of the tumor. PNETs account for 4% of all pediatric and adolescent cancers. Here we report a case of a peripheral primitive neuroectodermal tumor in a five-year-old boy. Two days before admission, he complained of multiple attacks of vomiting and one episode of hematemesis, associated with subjective fever, abdominal pain, and distention. He also complained of weight loss and bruises on his face and lower extremities for the last four weeks. Upon physical examination, there was hepatomegaly to the right iliac fossa. Abdominal ultrasound showed that the liver is hugely enlarged with heterogeneous echo texture and smooth borders. A computed tomography scan with contrast showed hepatomegaly to the right iliac fossa region with no focal lesion. Bone marrow aspiration and bone marrow biopsy showed heavy infiltration by monomorphic cells. Moreover, liver biopsy was done for this patient, and it showed metastatic undifferentiated neuroblastoma. Before the liver biopsy results, the patient deteriorated rapidly and dead. Therefore, peripheral primitive neuroectodermal tumors (pPNETs) should be considered in the differential diagnosis of liver masses in young patients to early diagnosis and treatment, and to increase the survival rate.

## Introduction

Primitive neuroectodermal tumors (PNETs) are a type of malignant tumor made up of small neuroectodermal-derived round cells that harm soft tissue and bone, with a wide range of clinical symptoms and histological commonalities [1]. They account for less than 1% of all sarcomas [2].

Based on the tissue of origin, Batsakis et al. categorized the primitive neuroectodermal tumor (PNET) family of malignancies into three following types: central nervous system (CNS), neuroblastoma, and peripheral primitive neuroectodermal tumors (pPNETs) [1].

These aggressive tumors, known as peripheral primitive neuroectodermal tumors (pPNETs), are frequently found in the thoracopulmonary region (Askin tumor), abdomen, and pelvis, and less frequently in the head and neck. Therefore, it invades nearby tissues, such as bone [1]. Stout identified pPNTEs for the first time in 1918 [3]. Only around 6% of them are extraosseous, but they typically start in the bones [4]. Clinically, PNETs present as abdominal pain, mass effect, and compression symptoms, such as abdominal distention and ascites [5].

Metastatic workup, including chest radiography, chest computerized tomography scanning, and a bone marrow biopsy is recommended in suspected cases due to the high frequency of metastatic illness at presentation [1]. The radiologic findings of pPNET are not clear and make the diagnosis difficult [6]. A multimodal strategy comprising chemotherapy, radiation, and surgery has been reported for treatment and prognosis [5]. Here we focus on peripheral primitive neuroectodermal tumors (pPNETs) and report a rare case among the pediatric population.

## Case presentation

A five-year-old Asian boy with no history of any medical illness, two days before admission, complained of multiple attacks of food containing vomiting and one episode of hematemesis, associated with subjective fever, abdominal pain, and abdominal distention. He also complained of weight loss and bruises on his face and lower extremities for the last four weeks. His physical examination revealed a distended abdomen, hepatomegaly to right iliac fossa, and splenomegaly.

Significant findings in initial laboratory investigations include the presence of low hemoglobin, RBCs, platelets, high direct bilirubin, and uric acid. In peripheral blood smear, WBCs showed mild left shifted granulocytes with some reactive and large granular lymphocytes and blast cells as well as marked thrombocytopenia. Moreover, RBCs showed marked normocytic anemia with prominent polychromatic cells and nucleated red blood cells.

The abdominal ultrasound showed that the liver is hugely enlarged with heterogeneous echo texture and smooth borders. No focal lesions or intrahepatic bile duct dilatation were seen. The portal vein is patent. The gallbladder is dilated and filled with thick turbid material associated with a thickened wall measuring 4 mm. Free fluid is noted in the perihepatic area and pelvis.

**Figure 1:**
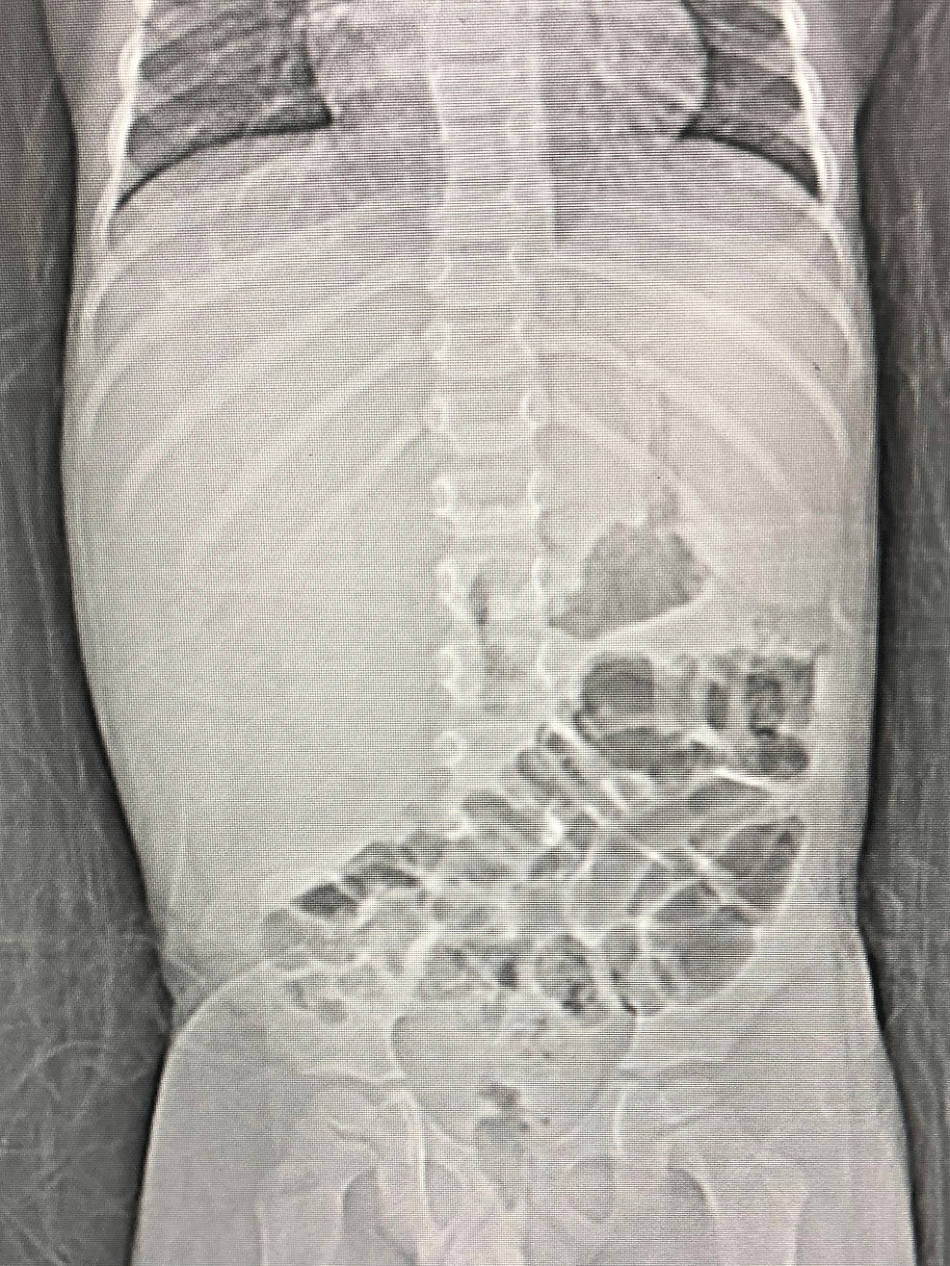
Abdominal and pelvic CT with contrast.

Moreover, abdominal and pelvic computed tomography (CT) scan with contrast was done and showed hepatomegaly, measuring 19.2 cm reaching down to right iliac fossa region with no focal lesion, and no intra- or extrahepatic cholestasis noted (Figure 1).

Bone marrow aspiration showed heavy infiltration by medium monomorphic cells either as individuals or in clusters. Normal hematopoietic cells are markedly suppressed, and many degenerated cells were seen. Bone marrow biopsy showed heavy infiltration by monomorphic cells which almost replaced the normal hematopoietic cells.

A liver biopsy was done and a microscopic examination revealed few hepatocytes infiltrated by a large neoplastic growth formed of solid nests and sheets of small to medium primitive and small round cells with, indiscernible/small amounts of cytoplasm, vague cytoplasmic borders, and numerous mitotic figures. The immunohistochemical profile of the patient is as follows: the malignant cells are strongly positive for - cluster of differentiation (CD)56, chromogranin, synaptophysin, S100, and vimentin. The malignant cells are weakly positive for - neuron-specific enolase (NSE) and ALK (faint and cytoplasmic), and the malignant cells are negative for - CK-PAN, CK7, CK20, HepPar-1, alpha-fetoprotein, SMA, desmin, myogenin, h-caldesmon, EMA, calponin, CD34, GFAP, P63, CD45, CD68, MelanA/Mart.

The patient was diagnosed with metastatic undifferentiated neuroblastoma. However, after the bone biopsy and aspiration results, and before the liver biopsy results, our patient deteriorated rapidly, became vitally unstable, and dead. The cause of death was mainly a delay in the diagnosis and no specific management or decision was made for him.

## Discussion

PNET is a highly malignant neoplasm and belongs to Ewing’s sarcoma family of tumors. They account for 4% of children and adolescent malignancies and are more common in teenagers and young adults, with a small male predominance [7].

They affect the paravertebral region, lower extremities, thorax, and abdomen. Abdominal involvement is rare and usually involves pancreas, stomach, and small intestine [5]. Peripheral primitive neuroectodermal tumors (pPNETs) account for 4-17% of all pediatric soft tissue tumors. These tumors are rare in African American children and Asian descent. Most worldwide cases are in white and Hispanic children and adolescents [1].

The basic treatment for peripheral PNET is multimodal therapy. Cases that can be operated on should undergo adjuvant chemoradiation in addition to the safest surgical excision possible. Tolerable individuals should receive moderately intense chemotherapy and radiotherapy to improve their prognosis [8].

People with an Ewing tumor had a five-year overall survival rate of 61%. The five-year survival rate is 81% in cases where the tumor is only discovered in the region where it first appeared (localized). The five-year survival rate is 67% if it has reached a nearby region (referred to as regional). The five-year survival rate is 38% if metastasis, or the tumor's distant dissemination was present at the time of diagnosis. According to age, this kind of cancer has an overall five-year survival rate of 76% for children under the age of 15 years and 59% for adolescents between the ages of 15 and 19 years [9].

Here we report a case of peripheral primitive neuroectodermal tumor pPNET in a five-year-old Asian boy. He presented with abdominal pain and distention. Abdominal and pelvic ultrasound and CT showed hepatomegaly. He also underwent aspiration and biopsy for the bone marrow, and liver biopsy to confirm the diagnosis. Our patient died with no specific management and no decision was made for him.

A similar case was reported by Montoya et al. in a four-year-old girl who presented with fever and abdominal pain. On her physical examination, she presented pale mucous membranes, multiple enlarged lymph nodes, and hepatomegaly. Total abdominal ultrasound showed hepatomegaly [5]. They did a chest CT of their patient, and it showed multiple subpleural, lung nodules compromising both lung fields. Moreover, they did abdominal magnetic resonance imaging (MRI), which showed diffuse hepatomegaly secondary to an intrahepatic, heterogeneous mass with necrotic areas. The lesion was biopsied for histological analysis. The pathology reported a malignant lesion. Immunohistochemical staining was positive for chromogranin, CD99, neuron-specific enolase (NSE), synaptophysin, and FLI-1. chemotherapy was initiated [5].

Sharma et al. reported a case of a four-year-old child who presented with abdominal pain and low-grade fever. There was no history of weight loss, but there was a loss of appetite. His physical examination showed moderate hepatomegaly and tenderness. Bone marrow aspiration and bone marrow biopsy examination were normal. Abdominal ultrasound revealed a large heterogeneously hypoechoic lesion in the liver with an exophytic lobulated cystic component with internal septations in the left lobe measuring 10.2×7×6.2 cm. Mild ascitic fluid was also noted [4].

Contrast-enhanced computed tomography (CECT) of the abdomen revealed a mass of size 110×100×60 mm, abutting the left lobe of the liver with likely loss of fat planes. The lesion showed areas of necrosis and calcification. Ultrasound-guided FNAC showed the presence of malignant round cells and a provisional diagnosis of hepatoblastoma was made. A liver biopsy and immunohistochemical assessment were done to confirm the diagnosis. Further investigations including a bone scan and PET scan were done to rule out any other site of involvement, and the patient started chemotherapy [4].

## Conclusions

Peripheral primitive neuroectodermal tumors (pPNETs) are rare and highly aggressive tumors in the pediatric population. Early diagnosis and treatment increase the survival rate, therefore, pPNETs should be considered in the differential diagnosis of liver masses in young patients to early diagnosis and management.
